# Supplementary material for: Provision of a liquefied petroleum gas cookstove and fuel during pregnancy and infancy and linear growth trajectories between birth and 12 months: evidence from the multi-center Household Air Pollution Intervention Network (HAPIN) trial
Source: medRxiv. 2025 Jun 6:2025.06.05.25329099. Preprint. [Version 1] doi: 10.1101/2025.06.05.25329099 (PMC12155024; doi:10.1101/2025.06.05.25329099)

medRxiv preprint doi: https://doi.org/10.1101/2025.06.05.25329099; this version posted June 6, 2025. The copyright holder for this preprint (which was not certified by peer review) is the author/funder, who has granted medRxiv a license to display the preprint in perpetuity.
It is made available under a CC-BY 4.0 International license .

SUPPLEMENTAL TABLES

medRxiv preprint doi: https://doi.org/10.1101/2025.06.05.25329099; this version posted June 6, 2025. The copyright holder for this preprint (which was not certified by peer review) is the author/funder, who has granted medRxiv a license to display the preprint in perpetuity.
It is made available under a CC-BY 4.0 International license .

Table #S1 Missing data for length-for-age z-score score measurements by timepoint, country site and intervention arm assignment for 2802 children whose mothers' completed participation in the HAPIN Trial

	Number (%) of missing LAZ measurements				
	**Birth**	**3 Months**	**6 Months**	**9 Months**	**12 Months**
**Total sample size at each time point**	2528	2093	2026	2036	2267
**Percent (%) missing at each time point**	10.84%	13.09%	13.52%	13.46%	12.09%
**Percent (%) missing by country site:**					
**Guatemala**	2.65%	3.20%	3.31%	3.29%	2.96%
**India**	4.43%	5.35%	5.53%	5.50%	4.94%
**Peru**	0.47%	0.57%	0.59%	0.59%	0.53%
**Rwanda**	3.28%	3.97%	4.10%	4.08%	3.66%
**Percent (%) missing by intervention arm assignment:**					
**Intervention**	5.78%	6.98%	7.21%	7.17%	6.44%
**Control**	5.06%	6.12%	6.32%	6.29%	5.65%
**Percent (%) missing by child sex:**					
**Female**	4.91%	5.92%	6.12%	6.09%	5.47%
**Male**	5.93%	7.17%	7.40%	7.37%	6.62%

Table #S2. Reasons for withdrawal or exiting the HAPIN Trial

135 mothers exited the HAPIN Trial before child's birth as a result of:<br>• 68 mothers experienced pregnancy loss<br>• 41 mothers voluntarily withdrew<br>• 21 mothers moved out from the catchment area<br>• 4 mothers were withdrawn by the study team<br>• 1 mother passed away
110 children exited the HAPIN Trial before study completion due to:<br>• 58 child deaths<br>• 26 children moved from catchment area<br>• 20 children were voluntarily withdrawn<br>• 1 child was lost to follow-up

Table #S3. Descriptive characteristics by HAPIN country site for 2802 children whose mothers' completed participation in the HAPIN Trial

medRxiv preprint doi: https://doi.org/10.1101/2025.06.05.25329099; this version posted June 6, 2025. The copyright holder for this preprint (which was not certified by peer review) is the author/funder, who has granted medRxiv a license to display the preprint in perpetuity.
It is made available under a CC-BY 4.0 International license .

	Guatemala	India	Peru	Rwanda
**Maternal height at baseline (Mean, SD)**	148.43 (5.31)	151.17 (5.67)	152.67 (4.48)	156.58 (5.95)
**Maternal age at baseline (Mean, SD)**	24.76 (4.43)	24.00 (3.77)	25.57 (4.54)	27.36 (4.38)
**Gestational age at baseline (Mean, SD)**	14.28 (3.04)	16.07 (2.97)	15.77 (3.35)	15.50 (2.79)
**Nulliparity (Yes) (N, %)**	209 (28.63%)	407 (56.61%)	229 (36.35%)	205 (28.35%)
**Infant sex (Female) (N, %)**	350 (47.95%)	327 (45.48%)	320 (50.79%)	354 (48.96%)
**Preterm birth (Yes) (N, %)**	41 (5.62%)	42 (5.84%)	25 (3.97%)	28 (3.87%)
**Number of people living in household (Mean, SD)**	5.18 (2.58)	3.81 (1.55)	4.55 (1.73)	3.50 (1.44)
**Socioeconomic index (Mean, SD)**	-0.44 (0.73)	0.97 (0.67)	0.46 (0.60)	-0.96 (0.86)
**Minimum dietary diversity (Mean, SD)**	3.05 (1.27)	2.87 (1.13)	5.03 (1.31)	2.92 (1.51)
**Household food insecurity (Mean, SD)**	1.17 (1.80)	0.49 (1.20)	1.38 (1.91)	2.50 (2.65)
**Length-for-age z-score at six-months (Mean, SD)**	-1.73 (1.04)	-1.11 (1.18)	-0.580 (0.984)	-0.862 (1.13)

Table #S4. Mean length-for-age z-score at each timepoint by intervention assignment and latent class trajectory for 2802 children whose mothers' completed participation in the HAPIN Trial

	Intervention Low	Intervention Medium	Intervention High	Control Low	Control Medium	Control High
Birth	-2.27	-1.15	-0.19	-2.42	-1.30	-0.42
3 Months	-2.79	-1.31	-0.12	-2.95	-1.42	-0.28
6 Months	-2.69	-1.36	-0.01	-2.91	-1.41	-0.12
9 Months	-2.78	-1.51	-0.08	-3.02	-1.54	-0.21
12 Months	-2.92	-1.65	-0.26	-3.10	-1.66	-0.41

Table #S5. Model fit statistics for latent class trajectories of length-for-age z-score by intervention arm for 2802 children whose mothers' completed participation in the HAPIN Trial

	One Class	Two Classes	Three Classes	Four Classes
Log-likelihood	-18458.19	-16829.77	-16124.40	-15736.55
AIC	36	33709.53	32312.79	31551.10
BIC	37059.27	33857.98	32502.81	31782.68

medRxiv preprint doi: https://doi.org/10.1101/2025.06.05.25329099; this version posted June 6, 2025. The copyright holder for this preprint (which was not certified by peer review) is the author/funder, who has granted medRxiv a license to display the preprint in perpetuity.
It is made available under a CC-BY 4.0 International license .

Adjusted BIC	37002.08	33778.55	32401.14	31658.76
Number of free parameters	18	25	32	39
Entropy	N/A	0.753	0.810	0.808

Table #S6. Model fit statistics for latent class trajectories of length-for-age z-score by country site, sex, timing of intervention and by sex within each country site

	HAPIN Country	Infant Sex	Timing of Intervention
Log-likelihood	-17791.34	-16074.45	-8091.96
AIC	35680.68	32206.89	16241.92
BIC	35971.65	32379.10	16394.25
aBIC	35815.96	32286.96	16302.13
Number of free parameters	49	29	29
Entropy	0.809	0.798	0.79

	Guatemala	India	Peru	Rwanda
Log-likelihood	-4145.982	-4130.948	-3170.245	-4186.355
AIC	8343.963	8313.896	6392.489	8424.71
BIC	8463.382	8432.92	6508.078	8543.878
aBIC	8380.824	8350.363	6425.531	8461.321
Number of free parameters	26	26	26	26
Entropy	0.827	0.707	0.787	0.859

*Akaike Information Criterion (AIC) and Bayesian Information Criterion (BIC) are methods for scoring and selecting a model. AIC puts more emphasis on model performance and selects more complex models whereas BIC penalizes complex models. The Adjusted Bayesian Information Criterion (aBIC) is the sample size-adjusted BIC.

medRxiv preprint doi: https://doi.org/10.1101/2025.06.05.25329099; this version posted June 6, 2025. The copyright holder for this preprint (which was not certified by peer review) is the author/funder, who has granted medRxiv a license to display the preprint in perpetuity.
It is made available under a CC-BY 4.0 International license .

Supplemental Figure 1. Path diagram of a longitudinal latent growth curve model. Observed variables are represented by squares and latent variables are represented by circles, with residuals represented by the small circles with an error term. The arrows between the latent variables, the intercept and slope, and the observed variables are fixed in advance. The factor loadings are fixed to 1 for the intercept latent variable and the factor loadings for the slope latent variable are fixed according to the change in time.

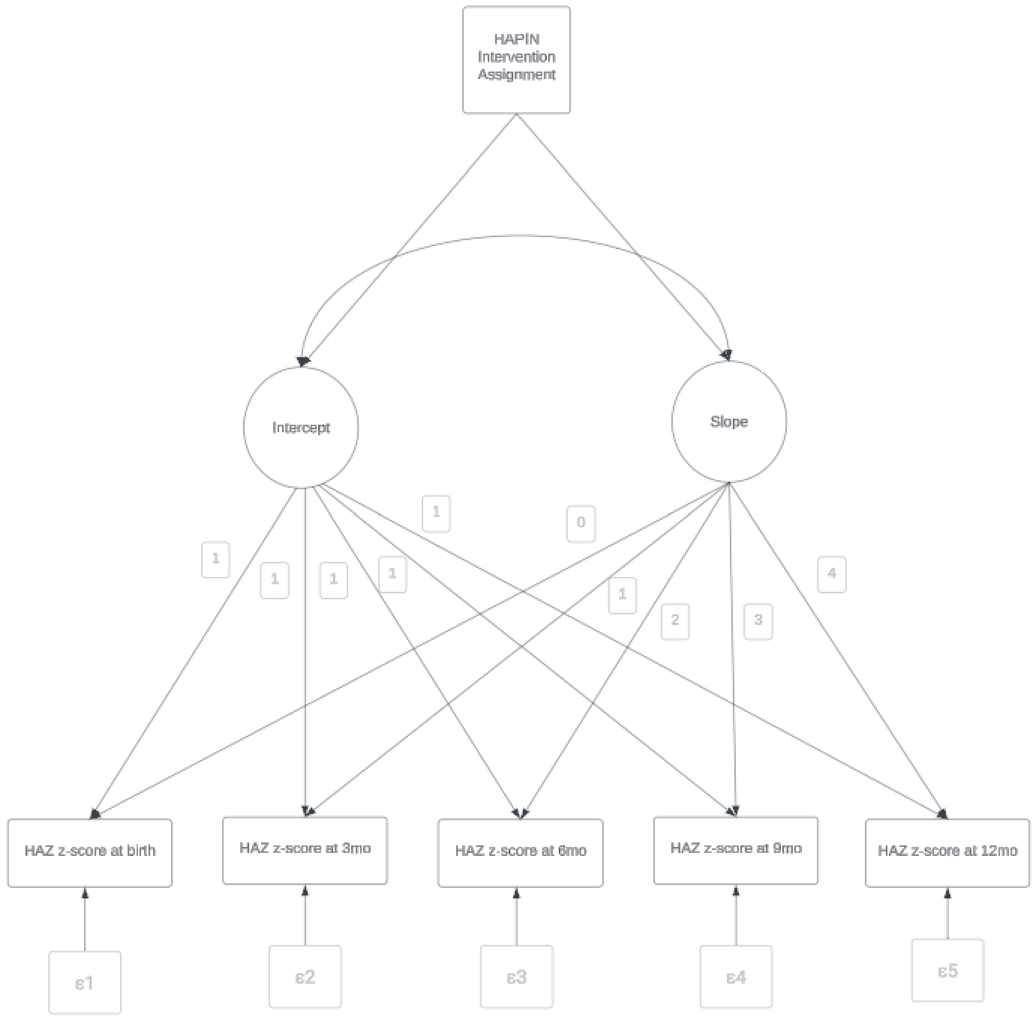

medRxiv preprint doi: https://doi.org/10.1101/2025.06.05.25329099; this version posted June 6, 2025. The copyright holder for this preprint (which was not certified by peer review) is the author/funder, who has granted medRxiv a license to display the preprint in perpetuity.
It is made available under a CC-BY 4.0 International license .

Supplemental Figure 2. Latent classes by intervention arm for intention-to-treat analysis

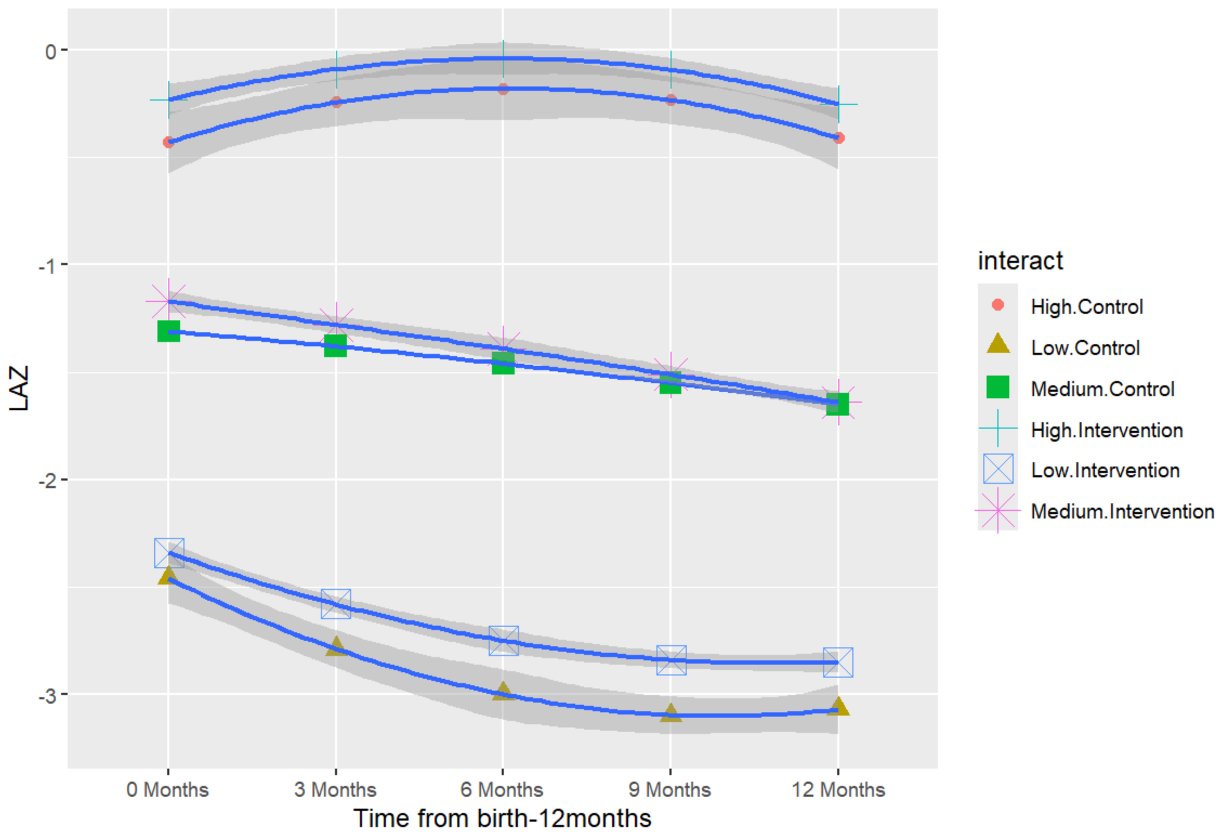

Supplemental Figure 3. Latent classes for timing of intervention delivery analysis

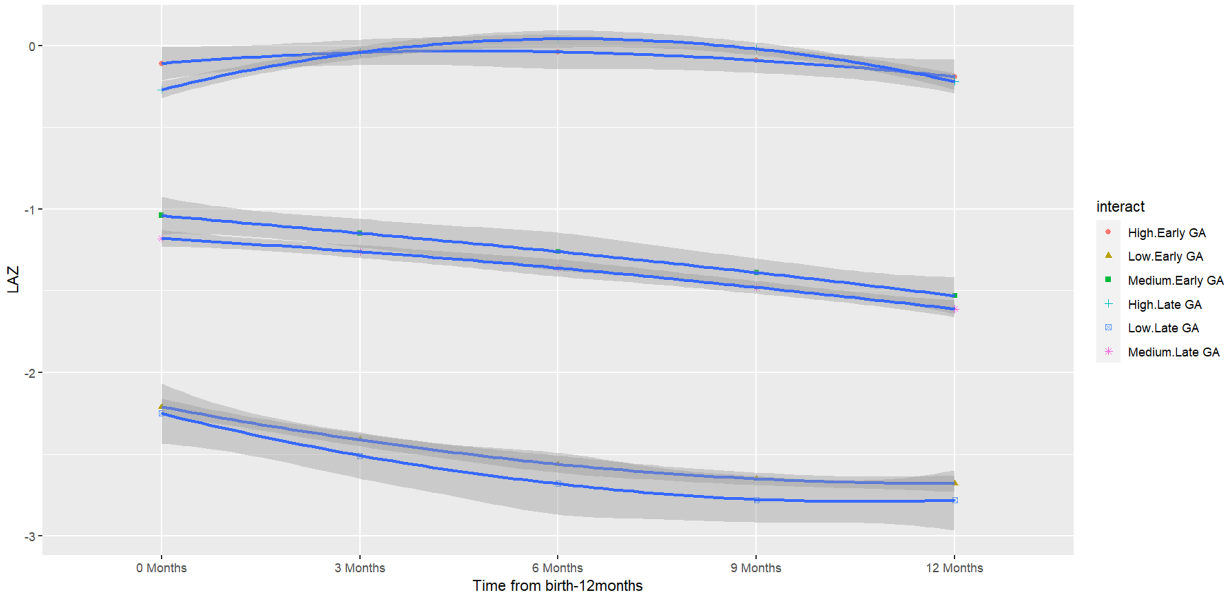

medRxiv preprint doi: https://doi.org/10.1101/2025.06.05.25329099; this version posted June 6, 2025. The copyright holder for this preprint (which was not certified by peer review) is the author/funder, who has granted medRxiv a license to display the preprint in perpetuity.
It is made available under a CC-BY 4.0 International license .

Supplemental Figure 4. Latent classes for sex analysis within Guatemala sample

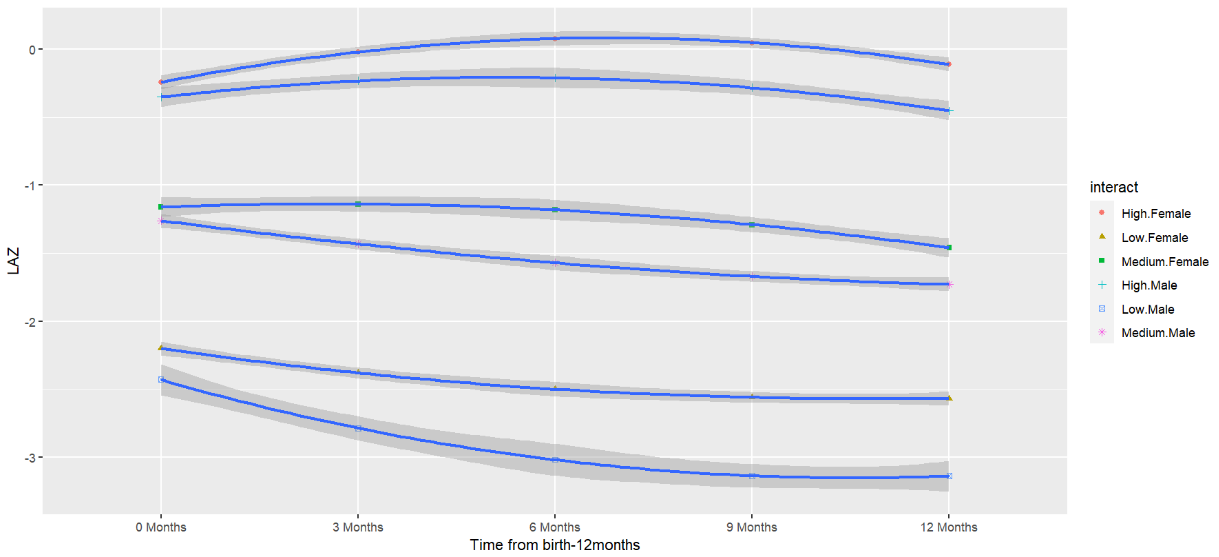

Supplement: Supplement 1 [file NIHPP2025.06.05.25329099v1-supplement-1.pdf]
